# Tape stripping method is useful for the quantification of antimicrobial peptides on the human skin surface including the stratum corneum

**DOI:** 10.1038/s41598-020-72111-6

**Published:** 2020-09-17

**Authors:** Shigeyuki Ono, Nobuhiko Eda, Takuya Mori, Atsuko Otsuka, Nobuhiro Nakamura, Yuto Inai, Noriyasu Ota, Takao Akama

**Affiliations:** 1 Biological Science Research, Kao Corporation, 2606 Akabane, Ichikai-machi, Haga-gun, Tochigi, 321-3497 Japan; 2 Japan Institute of Sports Sciences, Tokyo, Japan; 3 Waseda Institute for Sport Science, Saitama, Japan; 4 Faculty of Commerce, Yokohama College of Commerce, Kanagawa, Japan; 5 Graduate School of Sport Sciences, Waseda University, Saitama, Japan; 6 Faculty of Sport Sciences, Waseda University, Saitama, Japan

**Keywords:** Biochemistry, Biological techniques, Immunology, Health care

## Abstract

Antimicrobial peptides (AMPs) play an important role in innate immunity in human skin. It is known that AMPs mainly function in the stratum corneum. Therefore, AMP concentrations in the stratum corneum need to be precisely measured to clarify functional and physiological importance of AMPs in cutaneous defence. Tape stripping (TS) is a well-established method by which components in the stratum corneum can be collected. However, the usefulness of the TS method for measuring AMP concentration in human skin remains unclear. Therefore, we compared it with another popular method, skin rinsing, which had been established as a method for measuring AMP concentration in human skin. When investigated on healthy medial forearm using RNase 7, which is one of the typical AMPs, as an index, there was a significant positive correlation between RNase 7 concentrations measured by the TS method at adjacent forearm sites, demonstrating the reproducibility of the TS method. Next, a significant positive correlation was detected in RNase 7 concentrations measured using the TS and the skin rinsing method, indicating that the TS method is comparable to the skin rinsing method. Thus, we speculate that the TS method is useful for measuring AMP concentration in human skin.

## Introduction

Human skin is continuously exposed to xenobiotic substances, its own microbiota and microbial pathogens. Against intruding pathogens and opportunistic infections by commensal bacteria, human skin provides a chemical barrier based on the release of antimicrobial peptides, such as human beta-defensins (hBDs), S100A7 (psoriasin), and RNase 7, which are produced by epidermal keratinocytes, in addition to serving as a physical barrier. Therefore, the relationships between skin diseases and the expression of AMPs have been actively investigated. For example, it has been reported that secretion of S100A7 is enhanced in lesional skin of atopic dermatitis (AD) patients compared with non-lesional AD skin and normal controls^1^. Harder et al*.* also indicated enhanced secretion of RNase 7, S100A7, and hBD-2 in AD and psoriatic skin^2^. These reports suggest that AMPs in human skin play an important role in innate immunity against intruding pathogens^1,2^. Moreover, since AMPs are primarily produced by epidermal keratinocytes, it is assumed that the main region of action of AMPs is in the stratum corneum. Thus, AMP concentrations in the stratum corneum needs to be precisely measured to clarify the functional and physiological importance of AMPs in innate cutaneous host defence, particularly in healthy human skin.

To measure AMP concentration in human skin, the skin rinsing method is well-established and widely employed^1–3^. Briefly, skin-derived washing fluid is collected from subjects by rinsing the skin with a skin rinsing buffer composed of, for example, 900 µL of 10 mM sodium phosphate buffer (pH 7.4) containing 150 mM NaCl, while pipetting^1,2^ or stirring with a microtube homogenizer^3^. However, this method has the following limitations: (1) the rinsing conditions cannot be well controlled, and (2) the measured value (AMP concentration) is standardized by the sample collection area, which is not a physiological parameter. These points therefore imply that the skin rinsing method requires a certain skill level of the experimenter. On the other hand, tape stripping (TS)^4–9^ is a well-known, established method, in which components in the stratum corneum, such as ceramide, cholesterol, free fatty acid, and natural moisturizing factor (NMF), can be collected under mostly non-invasive conditions similar to those of the skin rinsing method. Moreover, it is recognized that the TS method is a technique that can be easily used, and that measurements made using the TS method (e.g., ceramide content) can be corrected by the total protein content evaluated from the other halves of tapes^6–9^. However, the usefulness of the TS method for measuring AMP concentration in human skin is not clear because few studies have evaluated AMP concentration using the TS method^10,11^. Thus, in the present study, to reveal the usefulness of the TS method, this method was compared with the more established skin rinsing method, which is used for measuring AMP concentration in human skin^1–3^.

Some AMPs are constitutively expressed in healthy human skin. Among the constitutively expressed AMPs, RNase 7 has been reported to be an important AMP expressed in human skin^12–15^ and appears to be expressed uniformly in the sebum film layer to the stratum corneum according to immunohistochemical analysis^2^. Therefore, it seems that RNase 7 is an AMP that can be examined without a large bias against either method. Accordingly, in the present study, the comparison of these methods was performed using RNase 7 concentration as an index.

## Results

**Figure 1:**
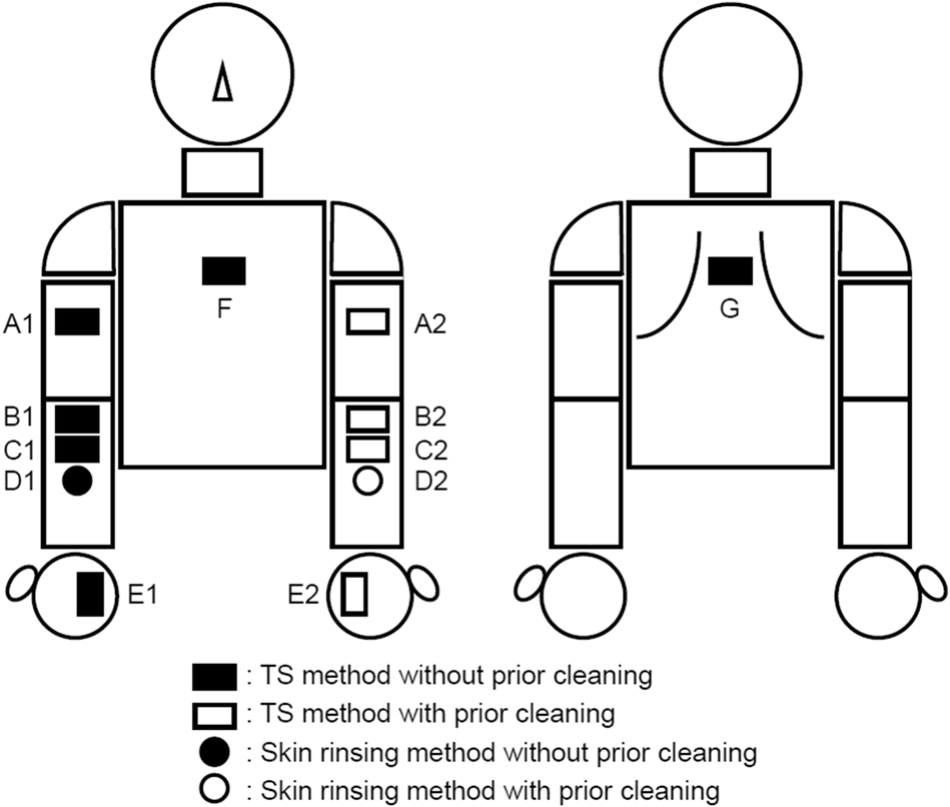
Sites and conditions for sample collection. Samples were obtained from the collection sites A1 to G at various skin sites using the TS or the skin rinsing method. Site A1: on the right medial upper arm; site B1, C1 and D1: on the right medial forearm; site E1: on the right palm; site A2: on the left medial upper arm; site B2, C2 and D2: on the left medial forearm; site E2: on the left palm; site F: on the thorax ventral; site G: on the thorax dorsal. At collection sites A1, B1, C1, E1, F and G, the TS method was performed under conditions of no prior cleaning of the collection site. At collection sites A2, B2, C2, and E2, the TS method was conducted under conditions of prior cleaning. At collection site D1, the skin rinsing method was performed under conditions of no prior cleaning. At collection site D2, the skin rinsing method was conducted under conditions of prior cleaning.

This study was performed under the following two conditions: (1) Under conditions of no prior cleaning of the AMP collection site with running water before sampling, and (2) Under conditions of prior cleaning of the AMP collection site with running water before sampling (see “Materials and Methods” and Fig. 1).

### Usefulness of the tape stripping (TS) method for measuring RNase 7 concentration under conditions of no prior cleaning of the collection site with running water

To elucidate the usefulness of the TS method for measuring RNase 7 concentration, both the reproducibility of the TS method and the relationship between the TS and the skin rinsing methods were investigated.

**Figure 2:**
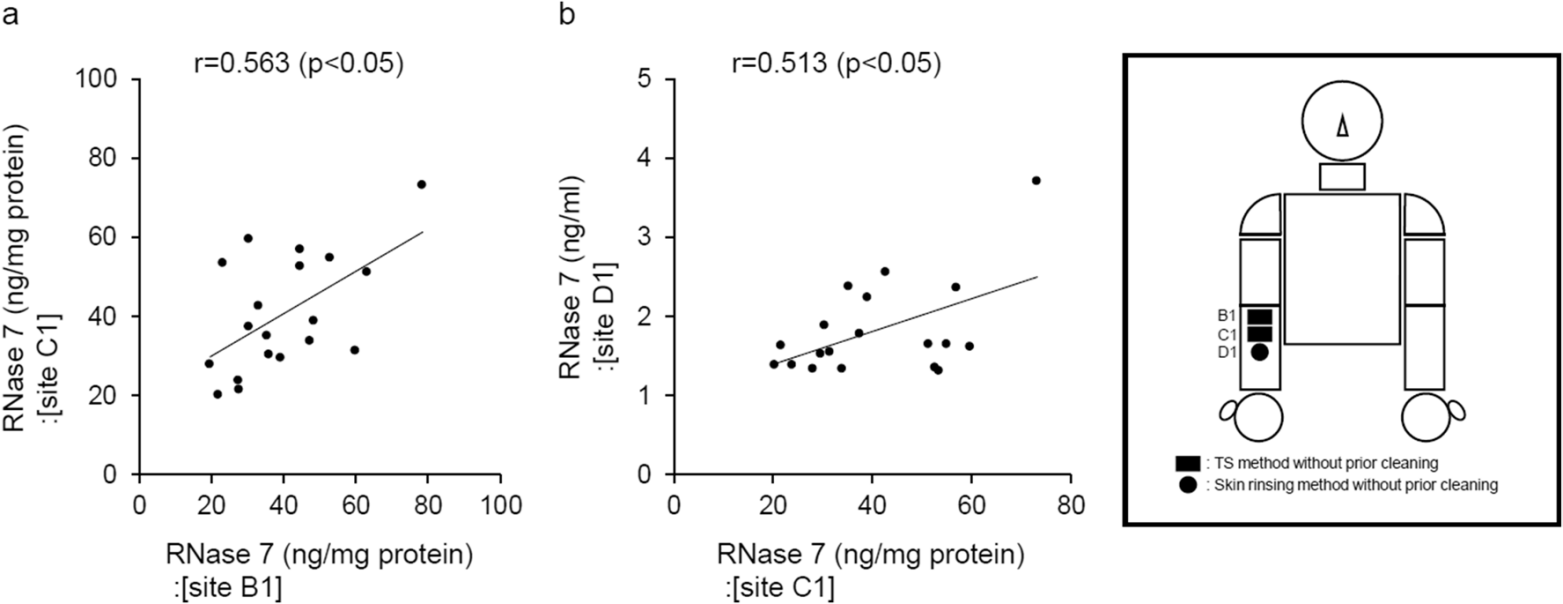
Examination of the usefulness of the TS method under conditions of no prior cleaning of the collection site. (**a**) For the examination of reproducibility, correlation analysis was performed between RNase 7 concentrations measured by the TS method at adjacent collection sites (site B1 vs. site C1). (**b**) For the examination of the relationship between both methods, correlation analysis was conducted between RNase 7 concentration measured by the TS method (site C1) and that measured by the skin rinsing method (site D1). (**a**,**b**) n = 19. Correlation was analyzed by Pearson’s correlation coefficient analysis.

Under conditions of no prior cleaning of the collection site with running water, the reproducibility of the TS method for measuring RNase 7 concentration was examined on the right medial forearm. RNase 7 concentrations at adjacent collection sites B1 and C1 were measured by the TS method, and then we performed correlation analysis of the RNase 7 concentrations obtained from these collection sites. A significant positive correlation was detected between RNase 7 concentrations at collection sites B1 and C1 (Fig. 2a).

To investigate the relationship between the TS method and the skin rinsing method under conditions of no prior cleaning of the collection site with running water, the RNase 7 concentration measured by the TS method at collection site C1 was compared with that measured by the skin rinsing method at collection site D1, which is adjacent to site C1 on the right medial forearm. There was a significant positive correlation between RNase 7 concentration measured by the TS method (site C1) and that measured by the skin rinsing method (site D1) (Fig. 2b).

### Usefulness of the TS method for measuring RNase 7 concentration under conditions of prior cleaning of the collection site with running water

**Figure 3:**
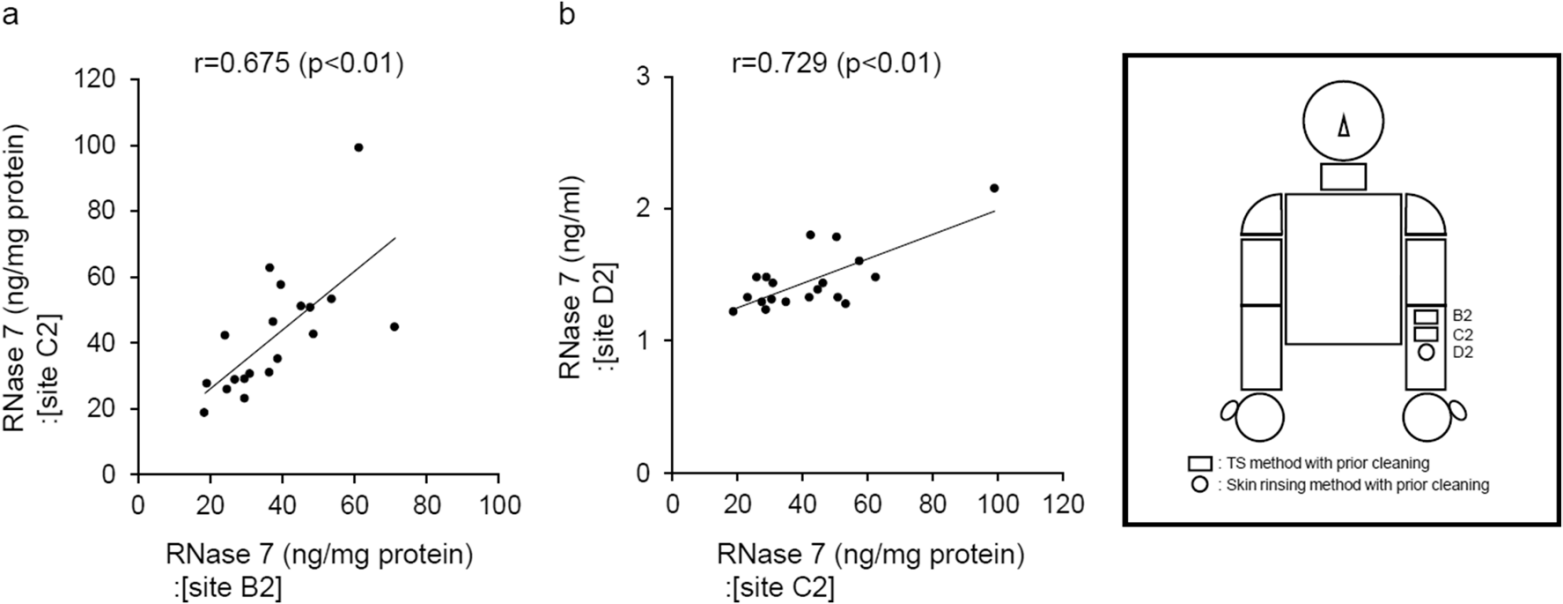
Examination of the usefulness of the TS method under conditions of prior cleaning of the collection site. (**a**) For the examination of reproducibility, correlation analysis was performed between RNase 7 concentrations measured by the TS method at adjacent collection sites (site B2 vs. site C2). (**b**) For the examination of the relationship between both methods, correlation analysis was conducted between RNase 7 concentration measured by the TS method (site C2) and that measured by the skin rinsing method (site D2). (**a**,**b**) n = 19. Correlation was analyzed by Pearson’s correlation coefficient analysis.

Similar to the above examination, the usefulness of the TS method for measuring RNase 7 concentration, under conditions of prior cleaning of the collection site with running water, was investigated on the left medial forearm. For the reproducibility of the TS method, a significant positive correlation was detected between the RNase 7 concentration measured at collection site B2 and that measured at adjacent collection site C2 (Fig. 3a). In addition, it was found that the correlation coefficient for the reproducibility was higher under conditions in which the collection site was pre-cleaned when compared to the conditions of no prior cleaning (Figs. 2a vs. 3a). On the other hand, for the relationship between the TS and the skin rinsing methods, there was a significant positive correlation between RNase 7 concentration measured by the TS method at collection site C2 and that measured by the skin rinsing method at collection site D2 which was adjacent to C2 (Fig. 3b). Moreover, like reproducibility, the correlation coefficient for the relationship between the TS and the skin rinsing methods was higher under conditions of prior cleaning of the collection site than under conditions of no prior cleaning of the collection site (Figs. 2b vs. 3b). Accordingly, these findings indicate that the usefulness (the reproducibility and the relationship to the skin rinsing method) of the TS method is elevated by prior cleaning of the collection site with running water. That is, it was found that the accuracy of the TS method is enhanced by prior cleaning.

### Comparison of RNase 7 concentrations measured by both the TS and the skin rinsing methods under conditions with and without prior cleaning of the collection site

**Figure 4:**
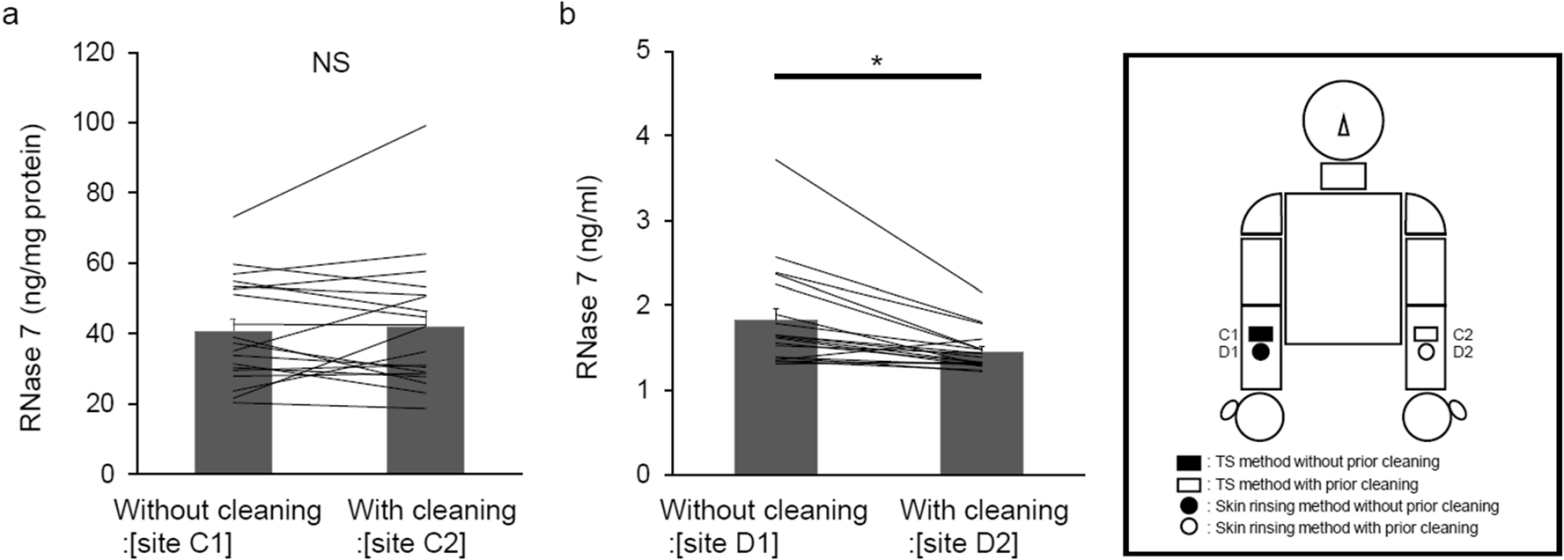
Examination of the characteristics of both the TS and the skin rinsing methods (**a**) Comparison of RNase 7 concentrations measured by the TS method under conditions with and without prior cleaning of the collection site (site C1 vs. site C2). (**b**) Comparison of RNase 7 concentrations measured by the skin rinsing method under conditions with and without prior cleaning of collection site (site D1 vs. site D2). (**a**,**b**) Mean ± SE. n = 19. Student’s *t*-test. *P < 0.05, NS: not significant.

To clarify the influence of prior cleaning of the collection site on the measurement values, RNase 7 concentrations measured by each method were compared under conditions both with and without prior cleaning of the collection site. In the TS method, there were no significant differences in RNase 7 concentration between conditions with and without prior cleaning of the collection site on the medial forearms (site C1 vs. C2) (Fig. 4a). On the contrary, in the skin rinsing method, RNase 7 concentration under conditions of prior cleaning of the collection site on the medial forearms was significantly lower than that without prior cleaning of the collection site (site D1 vs. D2) (Fig. 4b). Therefore, these results suggest that the extent to which RNase 7 concentration can be measured by the TS method is not largely influenced by the prior cleaning of the collection site, contrary to what was found for the skin rinsing method.

### Measurement of RNase 7 concentrations at various skin sites using the TS method

**Figure 5:**
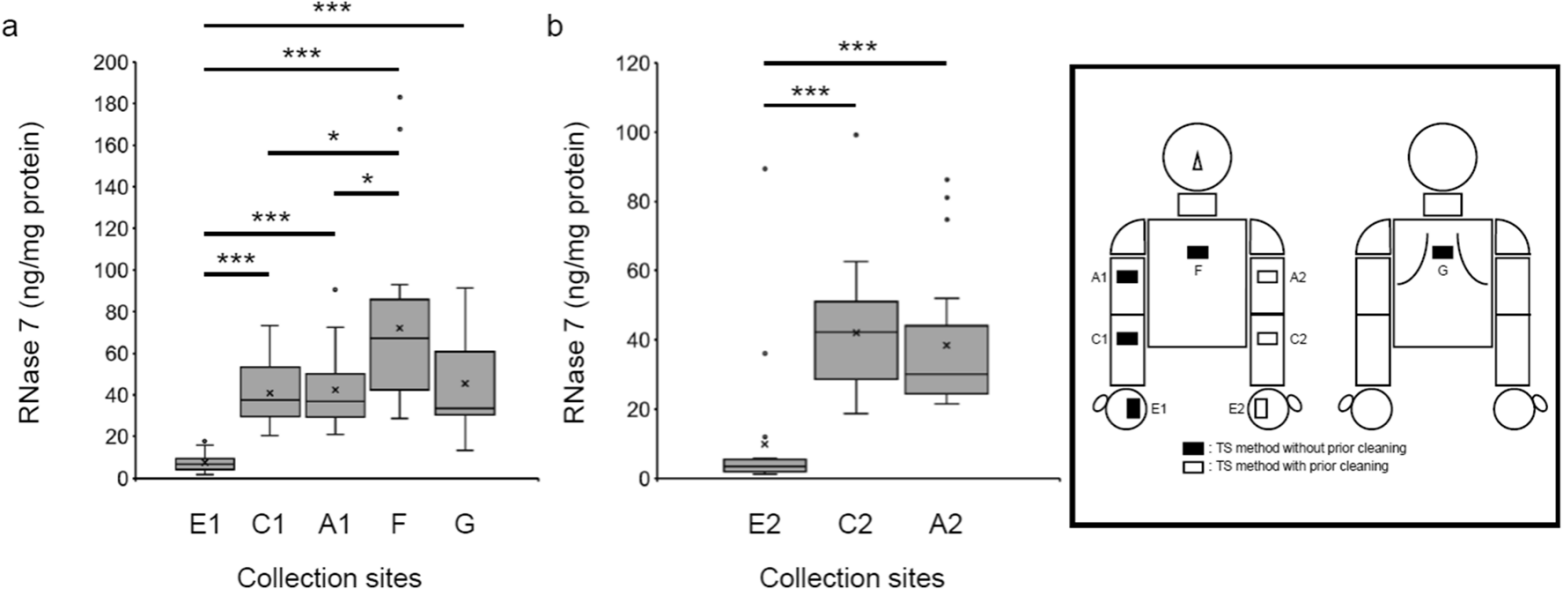
RNase 7 concentrations measured by the TS method at various skin sites. (**a**) Under conditions of no prior cleaning of the collection site, RNase 7 concentrations were measured at collection sites E1, C1, A1, F, and G. (**b**) Under conditions of prior cleaning of the collection site, RNase 7 concentrations were measured at collection sites E2, C2, and A2. (**a**,**b**) Median with inter-quartile range. n = 19. (**a**) Kruskal–Wallis test with Steel–Dwass test. *P < 0.05, ***P < 0.001. (**b**) One-way ANOVA with Tukey’s test. ***P < 0.001.

Since it was found that RNase 7 concentrations on the medial forearms can be measured by the TS method, we sought to measure RNase 7 concentrations at various skin sites using this method (Fig. 1). Under conditions of no prior cleaning of the collection site, RNase 7 concentration on the right palm (site E1) was significantly the lowest of the evaluated sites. In addition, RNase 7 concentration was significantly higher on the thoracic ventral (site F) than on the right medial upper arm (site A1) and the right medial forearm (site C1) (Fig. 5a). Similarly, under conditions of prior cleaning of the collection site, RNase 7 concentration was significantly lower on the left palm (site E2) than on either the medial upper arm (site A2) or the medial forearm (site C2) (Fig. 5b).

### Evaluation of the TS method for measuring S100A7 and hBD-3 concentrations under conditions of prior cleaning of the collection site

S100A7^1,16–18^ and hBD-3^19–21^ have also been reported to play an important role in innate cutaneous defence. Additionally, in this study, since both the reproducibility and the relationship to the skin rinsing method of the TS method were enhanced by prior cleaning of the collection site, we found that the accuracy of the TS method for measuring RNase 7 concentration is potentiated by prior cleaning. Therefore, we examined whether, under conditions of prior cleaning of the collection site, S100A7 and hBD-3 concentrations can also be measured by the TS method on the left medial forearm.

**Figure 6:**
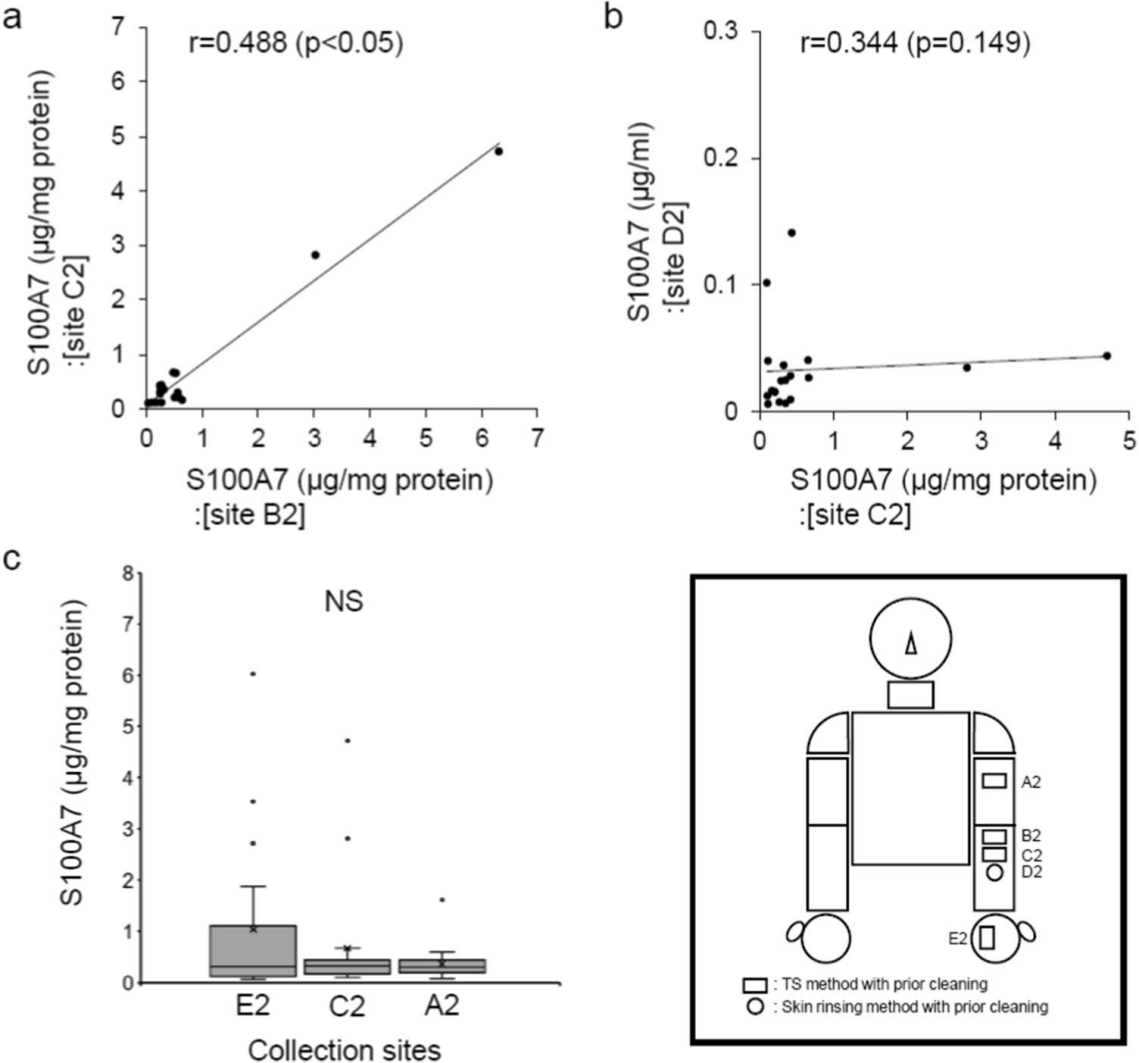
Evaluation of the TS method for measuring S100A7 concentration under conditions of prior cleaning of the collection site. (**a**) To examine reproducibility, correlation analysis was conducted between S100A7 concentrations measured by the TS method at adjacent collection sites (site B2 vs. site C2). (**b**) To examine the relationship of both methods, correlation analysis was conducted between S100A7 concentration measured by the TS method (site C2) and that measured by the skin rinsing method (site D2). (**c**) S100A7 concentrations were measured at collection sites E2, C2, and A2. (**a**,**b**) n = 19, Spearman’s correlation coefficient analysis. (**c**) Median with inter-quartile range. n = 19. Kruskal–Wallis test with Steel–Dwass test. *NS* not significant.

Individual differences in S100A7 concentrations, measured by the TS method, were large. That is, there were outliers in the data. Therefore, the correlation analysis for S100A7 concentrations was performed using Spearman’s correlation analysis (Fig. 6a,b). First, the reproducibility of the TS method for measuring S100A7 concentration was analyzed as described above. There was a significant positive correlation between S100A7 concentrations of adjacent collection sites B2 and C2, indicating the reproducibility of the TS method. On the contrary, there was no significant correlation between the S100A7 concentration measured by the TS method (site C2) and that measured by the skin rinsing method (site D2) (Fig. 6b). Moreover, there were no significant differences between the S100A7 concentrations measured by TS method at each of the three evaluated sites (medial upper arm, medial forearm, and palm) (Fig. 6c).

**Figure 7:**
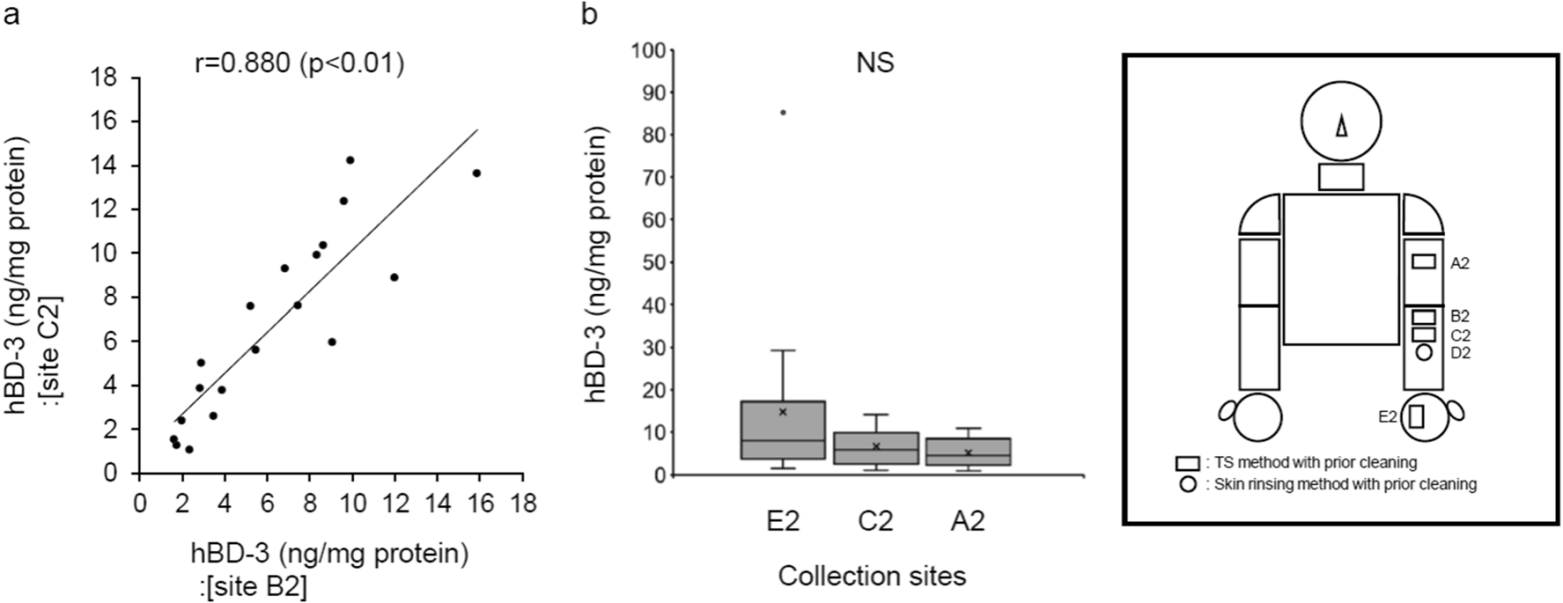
Evaluation of the TS method for measuring hBD-3 concentration under conditions of prior cleaning of the collection site. (**a**) Correlation analysis to evaluate the reproducibility of the TS method for measuring hBD-3 concentration (site B2 vs. site C2). (**b**) hBD-3 concentrations were measured at collection sites E2, C2, and A2. (**a**) n = 19. Pearson’s correlation coefficient analysis. (**b**) Median with inter-quartile range. E2: n = 16, C2 and A2: n = 19. Kruskal–Wallis test with Steel–Dwass test. *NS* not significant.

Second, similar to the examination of S100A7, we evaluated the reproducibility of the TS method for measuring hBD-3 concentration. There was a highly significant positive correlation between the hBD-3 concentrations of adjacent collection sites B2 and C2, indicating the reproducibility of the TS method (Fig. 7a). On the contrary, concentrations of hBD-3 on the left medial forearm (site D2), measured by the skin rinsing method, were detected only in 6 out of 19 subjects (data not shown). The hBD-3 concentrations of the remaining samples were below the detection limit of the ELISA. In addition, there were no significant differences between the hBD-3 concentrations measured by the TS method on the left medial upper arm, left medial forearm, and left palm (on the palm, hBD-3 concentrations in 3 out of 19 subjects were below the detection limit of the ELISA; n = 16) (Fig. 7b).

## Discussion

The TS method is well known to be useful for non-invasively collecting components in the stratum corneum, such as ceramides^4–8^ and NMF^22–24^. However, because the TS method is not practically employed in the field for measuring AMP concentration in human skin^10,11^, the usefulness of the TS method for measuring AMP concentration remains unclear. Therefore, in the present study, we sought to verify the usefulness of the TS method by comparison with another method that was previously established to measure AMP concentration in human skin, the skin rinsing method, using RNase 7 as an index. The main aims of this investigation are as follows: (1) If there is good reproducibility of RNase 7 concentrations measurements made by the TS method when examined at adjacent collection sites. and (2) If the result of the TS method when measuring RNase 7 concentration is comparable to that of the skin rinsing method, as examined at adjacent collection sites. To achieve this, we first investigated the reproducibility of the TS method. It was found that, regardless of the conditions with or without prior cleaning of the collection site, there was a significant positive correlation between RNase 7 concentrations that were measured in samples collected from adjacent sites of the medial forearm. These results imply that the TS method is a reproducible method for measuring RNase 7 concentration in human skin. We then examined the relationship between the two methods, the TS method and the skin rinsing method. In addition to reproducibility, regardless of the conditions with or without prior cleaning of the collection site, a significant positive correlation was detected between the RNase 7 concentrations measured by the TS method and the skin rinsing method, when evaluated at adjacent collection sites on the medial forearm. These results indicate that the TS method is comparable to the skin rinsing method in measuring RNase 7 concentration in human skin. Therefore, the usefulness of the TS method for measuring the concentration of RNase 7, which is a typical AMP in human skin^12–15^, was confirmed. These findings suggest that the TS method can be used as a means of measuring the concentration of RNase 7.

At present, the characteristics of each method for measuring AMP concentration are poorly understood. Therefore, we compared RNase 7 concentrations measured by each method under conditions with and without prior-cleaning of the collection site. It is usually thought that the sebum film layer is susceptible to cleaning with running water, and that, on the contrary, the stratum corneum is less susceptible to such cleaning. As shown in Fig. 4b, although the change in RNase 7 concentrations measured between under conditions with and without prior cleaning of the collection site were not very large, the prior cleaning step significantly reduced the RNase 7 concentrations measured by the skin rinsing method. These results indicate that the skin rinsing method mainly evaluates the RNase 7 concentrations in the sebum film layer and only partially evaluates that in the stratum corneum. On the other hand, the extent to which RNase 7 concentrations were measured by the TS method, was not largely influenced by the prior cleaning of the collection site (Fig. 4a). Thus, the TS method seems to measure the amount of RNase 7 present in both the sebum film layer and the stratum corneum. Moreover, RNase 7 concentrations measured by the TS method were about 20-fold higher than those measured by the skin rinsing method. Since the sebum film layer is thin, with a small volume, and tape stripping is a procedure for collecting the stratum corneum, it is suggested that under the TS method, the amount of RNase 7 existing in the stratum corneum primarily contributes to the detected RNase 7 concentration. Taken together, we found that each method measures the concentration (amount) of RNase 7 in different regions of the human epidermis.

Concentrations of AMP in the stratum corneum of both AD and healthy skin were previously measured using the TS method^10^. In that report^10^, AMP was extracted from the collection tapes with PBS under ultrasonic conditions at 0–4 °C. AMP concentration in the extract was measured using an ELISA kit. The total protein content, including amino acids in the same extract, was separately determined using a protein assay kit, and the AMP concentrations were corrected against this value. However, the conditions of extraction from the collection tapes in the previous study^10^ were similar to those used for analysing the NMF content^22–24^. Thus, it appears that the extract obtained with PBS mainly contains components that constitute the NMF in the stratum corneum, such as amino acids, but not the proteins that compose the stratum corneum. In addition, amino acids derived from the NMF are not extracted from the collection tapes by PBS alone^22–24^ and it is commonly believed that the amino acids that constitute the NMF display relatively large individual differences in the composition and quantity. Accordingly, it is suggested that the total protein content determined from an extract obtained with PBS alone might not be preferable as a correction factor. The total protein content derived from proteins that compose the stratum corneum is widely used to correct target measurements, such as ceramide concentration in human stratum corneum^6–9^, since it is generally believed that individual differences in the quality of the stratum corneum proteins are small. In the present study, half of each tape strip was used to extract AMP with PBS solution containing 1% BSA. Since the extract for measuring AMP concentration contained proteins (BSA), it cannot be used to measure the total protein content for the purpose of correcting the AMP concentrations measured by the TS method. Thus, we corrected the AMP concentration measurements against the total protein content in the extract obtained by processing the other half of each tape strips with an alkaline aqueous solution at 60 °C, which is a well-accepted method for extracting the stratum corneum proteins^6–9^. Accordingly, the total protein content that reflects the amount of protein composing the stratum corneum may be a more appropriate correction factor. Further studies in the future will reinforce the validity of the total protein content as a correction factor.

In the present study, we showed that the correlation coefficients related to the usefulness (the reproducibility and the relationship to the skin rinsing method) of the TS method for measuring RNase 7 concentrations were higher under conditions with prior cleaning of the collection site than under conditions without prior cleaning. Namely, the accuracy of the TS method was augmented by prior cleaning of the collection site, suggesting that conditions under which the collection sites are cleaned prior to sampling are more suitable for measuring RNase 7 concentrations in human skin. Consequently, it is recommended that the TS method is performed on cleaned human skin such as prior cleaning with running water before sampling.

In Figs. 2 and 3, we demonstrated the usefulness of the TS method for measuring RNase 7 concentration. However, the correlation coefficients, particularly in Fig. 2, were not necessarily large. In the present study, one half of each tape strip was used for measuring RNase 7 concentration, while the other half was used for total protein content analysis. The homogeneity of the stratum corneum collected on the tape during the tape stripping procedure is reported to be uneven^25^. This might be one of the reasons why high correlation coefficients were not obtained. Therefore, it seems that when the TS method is applied for measuring AMP concentration in human skin for precise evaluation of the AMP concentration, the heterogeneity of the stratum corneum collected on the tape should be considered. To reduce this risk, the use of a large piece of tape has been proposed^25^. Since we used relatively large pieces of tape in this study, it is likely that this helped to ensure the homogeneity of the stratum corneum on the tape. Moreover, as shown above, we found that the accuracy of the TS method for measuring RNase 7 concentration was potentiated by prior cleaning of the collection site. This finding suggests that the homogeneity of the stratum corneum on the tape may be improved by increasing the adhesion of the tape to the skin. Accordingly, prior cleaning of the collection site may also be an important factor in ensuring the homogeneity of the stratum corneum collected on the tape.

In human skin, AMPs are primarily produced by keratinocytes. As AMPs are in general thought to function in the lamellar membrane bilayers of the stratum corneum, it seems that AMPs contained in corneocytes of the stratum corneum are not available for the innate immune defense of human skin under physiological conditions. Under our extraction conditions from the collection tapes, it is presumed that AMPs in corneocytes may be also collected into the extract solution. In this case, if the corneocytes containing AMPs exist as a large pool, AMP concentrations measured using the TS method might be overestimated under physiological conditions. It has been reported that AMPs produced in keratinocytes, such as LL-37, hBD-2 and hBD-3, are stored in the lamellar bodies, as are ceramides, and released through these vesicles into the extracellular spaces of the stratum corneum (intercellular membrane bilayers) during the differentiation process of keratinocytes^26–28^. Köten et al*.* demonstrated in the cell culture experiments using primary keratinocytes that the majority of RNase 7 is released from the cells^29^. Moreover, it has been recently reported that S100A7 is sustainably secreted from both proliferating and differentiated keratinocytes^30^. Therefore, most AMPs produced in keratinocytes appear to be released from cells under normal conditions, suggesting that in healthy human skin, the level of AMPs in corneocytes formed after differentiation of keratinocytes may be low and at least, the possibility is high that there may be no large pool of AMPs by corneocytes. Thus, even if AMPs are present in corneocytes, it is possible that their contribution to the AMP concentrations measured by the TS method is small. Taken together, it seems likely that the AMP concentrations measured using the TS method investigated in the present study represent valid measurements under physiological conditions in healthy human skin. However, it remains obscure whether there are indeed few AMPs in corneocytes of healthy human skin. Therefore, in order to establish the TS method as a means for more precisely measuring AMP concentrations under physiological conditions in healthy human skin, it will be important to investigate in the future whether AMPs are present in corneocytes and, if so, how only AMPs in the lamellar membrane bilayer can be extracted from collection tapes and whether AMPs in corneocytes are involved in the innate immune defense of human skin.

We also investigated the usefulness of the TS method for measuring S100A7 and hBD-3, which are other typical AMPs found in human skin, under conditions of prior cleaning of the collection site. Both S100A7 and hBD-3 could be measured by the TS method with significant reproducibility (Figs. 6a and 7a). However, there was no significant positive correlation between S100A7 concentrations measured by the TS method and those measured by the skin rinsing method (Fig. 6b). If S100A7 exists uniformly in the sebum film layer to the stratum corneum, it would be expected that, as found for RNase 7, S100A7 concentration measured by the TS method would positively correlate with that measured by the skin rinsing method. Thus, S100A7 might not exist uniformly throughout the sebum film layer and the stratum corneum, unlike RNase 7. Although it has been reported that S100A7 can be sufficiently detected by the skin rinsing method in human skin^1,2,16^, immunostaining levels of S100A7 throughout the sebum film layer and the stratum corneum of healthy human skin seem to be obscure. Further studies are needed to clarify the locations and distribution of S100A7 in the human epidermis.

From results obtained by the skin rinsing method, it is believed that hBD-3 is an inducible AMP that is found only in inflamed human skin^2,31^. Consistent with this, we showed that most of the concentrations of hBD-3 measured by the skin rinsing method were below the detection limit of the ELISA, indicating that hBD-3 is not constitutively expressed in healthy human skin when evaluated by the skin rinsing method. However, it has been reported that hBD-3 was expressed in the stratum corneum of healthy human skin in 40% of the biopsies analyzed by immunohistochemical study^32^ and that hBD-3 was detected in healthy controls using the TS method^10^. In the present study, hBD-3 concentration in healthy human skin was measured using the TS method with high reproducibility (Fig. 7a). As mentioned above, our study revealed that the TS method and the skin rinsing method mainly measured AMP concentration in the stratum corneum and the sebum film layer, respectively. Therefore, our results suggest that hBD-3 primarily exists in the stratum corneum, but not in the sebum film layer of healthy human skin. Notably, hBD-3, which appears to be constitutively expressed in the human epidermis, can only be properly detected by the TS method, and as such, in the case of hBD-3, it is not possible to examine the correlation between both methods. From the above results, the usefulness of the TS method for measuring S100A7 and hBD-3 concentrations in human skin was also confirmed. Accordingly, we can conclude that the TS method can be used to measure the concentrations of typical AMPs expressed in human skin, such as RNase 7, S100A7 and hBD-3.

S100A7 has been reported to be constitutively expressed in human skin in studies using a skin rinsing method^1,2,16^. In the present study, we identified S100A7 as being present on the medial forearm of healthy humans using both the TS and skin rinsing methods (Fig. 6). On the contrary, Clausen et al. reported that S100A7 was only sporadically detected at the same skin site of healthy humans using the TS method^10^. Thus, there was a difference between our TS method and that used by Clausen et al*.* in terms of the ability to detect S100A7. Clausen et al*.* used PBS alone to extract AMPs from the collection tapes. However, in our TS method, PBS containing 1% BSA was used as the extraction solution. It is well known that BSA is added to stabilize small amounts of protein contained in an aqueous solution. This might be the reason why the TS method used by Clausen et al*.* could not sufficiently detect S100A7 in healthy human skin. Hence, we consider that S100A7 is a constitutively expressed AMP in healthy human skin and can be detected using the TS method.

In the present study, we conducted a preliminary study on the site differences in AMP expression levels in human skin (Figs. 5, 6c and 7b). In the TS method, RNase 7 concentrations were in the order of medial forearm ≈ medial upper arm > palm (Fig. 5b). On the other hand, it has been reported that when the skin rinsing method with pipetting was employed, RNase 7 concentrations at the same skin sites as in Fig. 5b were in the order of medial forearm > palm > medial upper arm^29,33^. The reason for these differences is unclear at present. We showed that in RNase 7 concentrations on the medial forearm, the results of the TS method were in good agreement with those of the skin rinsing method with stirring by a microtube homogenizer used in this study. Therefore, there may be differences regarding extraction efficiency between the skin rinsing method with stirring by a microtube homogenizer and the skin rinsing method with pipetting. A further reason might be that the TS method and the skin rinsing method each measure the amount of AMP in different layers of the human epidermis with discrete contributions, as described above. Because the TS method has good reproducibility, in the future, it would be interesting to comprehensively re-evaluate the skin site differences in the expression levels of various AMPs using this method. On the other hand, we do not currently know of any physiological reasons for the skin site differences in RNase 7 expression levels. Therefore, we think that further studies are needed to elucidate physiological significances of the skin site differences in RNase 7 expression levels.

In the present study, it was shown that the skin rinsing method mainly measured AMP concentration in the sebum film layer, while the TS method primarily measured that in the stratum corneum. This implies that the skin rinsing method, but not the TS method, evaluates the concentration of AMP that have accumulated at the skin surface, namely the free AMP concentration of the skin surface. It has been reported that although high-intensity endurance exercise depresses the immune barrier, such as by altering the concentration of secretory immunoglobulin A on the skin surface, in order to complement the compromised skin barrier, hBD-2 is secreted to the skin surface^3^. This report is likely to suggest the importance of free (and/or transient) AMPs accumulated at the skin surface. Therefore, in the case of investigating the skin barrier of free (and/or transient) AMPs present at the skin surface, for example, the skin rinsing method may be one of the appropriate means to do so. On the other hand, as AMPs are largely produced by epidermal keratinocytes, it is presumed that AMPs in the stratum corneum constitutively play an important role in providing a chemical barrier against intruding pathogens and opportunistic infections by commensal bacteria, along with the physical barrier function composed of both the corneocytes and the intercellular membrane bilayers containing ceramides. To clarify the constitutive skin barrier functions of AMPs, in particular in healthy human skin, AMP concentrations in the stratum corneum will need to be precisely measured. In this study, we showed that the TS method primarily measured AMP concentrations in the stratum corneum. Therefore, in the case of investigating the constitutive skin barrier functions of AMPs in the stratum corneum, for example, the TS method may be one of the appropriate means to do so.

In summary, our data suggest that the good reproducible TS method is a useful means for measuring the concentrations of AMPs present in human skin, such as RNase 7, S100A7 and hBD-3. Additionally, the TS method mainly measures AMP concentrations (amounts) in the stratum corneum which are predicted to function as a first line of defence against invading microorganisms.

## Materials and methods

### Subjects

Nineteen healthy adult males (aged 24.9 ± 2.2 years; height 173.3 ± 5.1 cm; body mass 70.7 ± 9.1 kg; body fat percentage 18.1 ± 4.5%; and body mass index 23.6 ± 3.0 kg·m^−2^) were recruited for the study by snowball sampling. All subjects had passed a complete medical examination within the preceding year and were given a detailed explanation of risks, stress, and the potential benefits of the study before they signed an informed consent form. The study was approved by the Ethics Review Committees of Waseda University and Kao Corporation. Experiments were conducted in accordance with the guidelines of the Declaration of Helsinki Principles.

### Conditions of sample collection for measuring AMP concentrations at targeted skin sites

Before the start of sample collection to measure AMP concentrations at various skin sites (Fig. 1), to remove dirt present on the skin, only the medial surface of the left arm (left medial upper arm, left medial forearm and left palm) of each subject was washed with running water from a shower head for 30 s. Specifically, the skin surface of the subject was washed by gently stroking it with the palm of a gloved experimenter’s hand at a constant speed. These skin sites were the AMP collection sites under conditions of prior cleaning with running water (site A2, B2, C2, D2 and E2). The remaining skin sites were the AMP collection sites under conditions of no prior cleaning with running water (site A1, B1, C1, D1, E1, F and G). Thereafter, the subjects were moved to a climate-controlled room at 20 ± 2 °C and 40 ± 5% relative humidity. After acclimating the controlled environment for 15 min, samples for measuring AMP concentrations at targeted skin sites were collected by the TS method or the skin rinsing method.

### Sample collection by the TS method

To collect samples for measuring AMP concentrations at various skin sites by the TS method, tape stripping was performed by pressing an acryl film tape with an area of 25 mm × 50 mm (465#40; Teraoka Seisakusho, Tokyo, Japan) to the targeted skin surface. In this study, five consecutive tape strips were obtained from each skin collection site. Each tape strip was divided into two half-strips excluding 5 mm from the long axis end of tape: one half was used for measuring AMP concentration and the other half for total protein content analysis. The halves of tape strips for measuring AMP concentration were further cut into 1/8 size pieces. All pieces were immersed in 600 µL sodium phosphate buffer containing 1 mM ethylenediaminetetraacetic acid disodium salt, 2-hydrate [EDTA-2Na] (Sigma Aldrich, St. Louis, MO, USA), 1% (w/v) bovine serum albumin (Sigma Aldrich), and 0.05% (v/v) Tween 20 (Promega, Madison, WI, USA), followed by ultrasonic treatment for 15 min (Bioruptor UCW-310; Cosmo Bio, Tokyo, Japan) at 4 °C. After removing the pieces of tape, the extracted samples were centrifuged (5,000 rpm, 15 min, 4 °C) and supernatants were stored at − 80 °C until further processing.

### Sample collection by the skin rinsing method

To collect samples for measuring AMP concentrations on the medial forearms by the skin rinsing method, one end of a polypropylene tube (a cylindrical guide) with open ends on both sides (Centrifuge Tubes; AGC Techno Glass, Chiba, Japan) was placed on the targeted skin surface. The same solution as that used in sample collection by the TS method (1 mL) was added into the cylindrical guide and the solution in the cylindrical guide was stirred at 9,000 rpm with a microtube homogenizer (23 M; As One, Osaka, Japan) for 60 s on the skin surface^3^. Thereafter, the solution in the cylindrical guide was recovered using a pipette, as a skin rinsing sample. The skin rinsing samples were centrifuged (5,000 rpm, 15 min, 4 °C) and supernatants were stored at − 80 °C until further processing.

### Measurement of AMP concentrations in collection samples

AMP concentrations in collection samples were measured using commercially available standard sandwich ELISA kits. RNase 7 concentrations were analyzed using an ELISA kit with a measurable concentration range of 78.1 to 5,000 pg/mL (HK371, Hycult Biotech, Wayne, PA, USA). S100A7 concentrations were determined using an ELISA kit with a measurable concentration range of 0.12 to 90 ng/mL (CY-8073, CycLex Co., Nagano, Japan). hBD-3 concentrations were analyzed using an ELISA kit with a measurable concentration range of 0.063 to 4 ng/mL (EK-072-38, Phoenix Pharmaceuticals, Burlingame, CA, USA).

### Total protein content analysis

The other halves of the tape strips were also further cut into 1/8 pieces. All pieces were immersed in 1 mL alkaline aqueous solution [0.1 N NaOH (Wako, Osaka, Japan), 1% (w/v) sodium dodecyl sulphate (Wako, Osaka, Japan)] and then incubated at 60 °C for 2.5 h. After incubation, the solution was neutralized with 1 N HCl (Wako, Osaka, Japan). The total protein content in each sample solution was measured by a BCA kit (Thermo Scientific, Rockford, IL, USA) using bovine serum albumin as a protein standard. The AMP concentrations measured using the TS method were corrected with the total protein content.

### Statistical analysis

Data are presented as the mean ± standard error (SE) or the median with inter-quartile range, depending on the distribution of data. Statistical analysis was performed using IBM SPSS statistics version 24 (IBM Corp., Armonk, NY, USA) and KyPlot 5.0 (Kyenslab Inc., Tokyo, Japan). In the statistical analysis, data were analyzed for normal distribution using Kolmogorov–Smirnov test and for homoscedasticity using Bartlett test. One-way ANOVA or Kruskal–Wallis test (one-way non-parametric ANOVA) was used to evaluate differences between multiple groups. A parametric (Tukey’s test) or non-parametric (Steel–Dwass test) post hoc multiple comparison test was performed to evaluate differences between the groups where appropriate. Differences between two groups were compared using Student’s *t*-test. Correlations were also examined by Pearson’s or Spearman’s correlation coefficient analysis. P < 0.05 was considered statistically significant.
